# Experimental rodent models of cardiorenal syndrome types 3 and 4: Insights and clinical relevance (Review)

**DOI:** 10.3892/ijmm.2026.5881

**Published:** 2026-06-04

**Authors:** Stefanny M. Figueroa, Siyi Huang, Javier Reyes-Osorio, Jean-Jacques Boffa, Cristián A. Amador, Christos E. Chadjichristos, Louis Boutin

**Affiliations:** 1 INSERM UMRS 1155-Common and Rare Kidney Diseases, Tenon Hospital, Faculty of Medicine, Sorbonne University, 75020 Paris, France; 2 Facultad de Ciencias, Universidad San Sebastián, Santiago 7510157, Chile; 3 Sorbonne Université, INSERM UMRS 1155, AP-HP, Department of Nephrology, Hôpital Tenon, 75020 Paris, France; 4 INSERM, UMR 942, MASCOT: Cardiovascular Marker in Stress Condition, Lariboisière Hospital, Université de Paris Cité, 75010 Paris, France; 5 Department of Anaesthesiology and Intensive Care, Hôpital Européen Georges Pompidou, AP-HP, 75015 Paris, France; 6 Investigation Network Initiative-Cardiovascular and Renal Clinical Trialists Network, 54500 Vandoeuvre-lés-Nancy, France

**Keywords:** cardio-renal syndrome type 3 and 4, acute kidney injury, chronic kidney disease, cardiac dysfunction, experimental models, clinical applications

## Abstract

Cardiorenal syndrome (CRS) encompasses the bidirectional and complex interaction between cardiac and renal dysfunction. The present review focuses on CRS types 3 and 4, highlighting the negative effects of acute kidney injury and chronic kidney disease, respectively, on cardiac function. The present review focuses on pathophysiological mechanisms, associating renal impairment with cardiovascular events, paying particular attention to systemic inflammation, oxidative stress, endothelial damage and neurohormonal activation. From fundamental science to clinical applications, the investigation of CRS remains a challenge. In this context, some experimental models mimicking CRS types 3 and 4 have been used, including 5/6 nephrectomy, unilateral ureteral obstruction, renal ischemia-reperfusion and adenine- or cisplatin-induced kidney injury. While these models are valuable for studying such disease mechanisms, their limitations in mimicking human pathophysiology are discussed, and their strengths and weaknesses are critically addressed. Advancing and refining preclinical models should be prioritized in future research to enhance clinical relevance and accelerate the development of targeted therapies for CRS.

## 1. Introduction

In previous years, the relationship between kidney failure and heart disease has gained more attention and is now a major focus of research in the scientific community (1-4). Extensive research supports the idea of a bidirectional relationship, demonstrating that renal failure might affect the progression and development of cardiac diseases, and vice versa (3,5). This pathophysiological concept is known as cardiorenal syndrome (CRS) and consists of five distinct subtypes based on the organ of origin (heart or kidney) and the rate of progression (acute or chronic). Type 1 CRS is characterized by an acute decline in cardiac function, causing acute renal damage; type 2 leads to chronic cardiac abnormalities, resulting in chronic renal damage; type 3 leads to acute renal damage causing acute cardiac dysfunction; type 4 leads to chronic renal damage, resulting in chronic cardiac dysfunction; and finally type 5 involves systemic diseases causing simultaneous cardiac and renal damage (Table I) (1,3,5).

The growing incidence of both acute kidney injury (AKI) (6,7) and chronic kidney disease (CKD) (8,9) has driven renewed and sustained scientific attention. Based on a cohort of patients surviving hospitalization following AKI, ~42% were readmitted ≤30 days, mainly due to congestive heart failure or myocardial infarction (10). Similarly, Go *et al* (11) found that patients with AKI accounted for 44% of heart failure readmissions during the first year post-hospital discharge. Furthermore, another study by Odutayo *et al* (12) showed that patients exhibited an 86% risk of cardiovascular (CV) mortality following AKI. All the aforementioned studies highlight that patients with AKI face an elevated risk of both short- and long-term CV events.

Development of CKD is supported by recurrent episodes of AKI (13-15), and increases the risk of CV morbidity and mortality (16-19). Data from the Kaiser Permanente Renal Registry show an appreciable rise in adjusted CV events risk with decreasing eGFR. Indeed, a 43% rise in risk was observed among persons with eGFR ranging from 45 to 59 ml/min/1.72 m^2^, while a 343% increase in risk was recorded among those with eGFR <15 ml/min/1.72 m^2^ (20). In established CKD, the incidence of *de novo* heart failure (HF) is between 17 and 21% (21). Patients with haemodialysis exhibited a higher probability of HF compared with those undergoing other therapeutic modalities. The onset of HF is influenced by the severity of CKD and the type of kidney replacement therapy, including kidney transplantation (22). The increasing prevalence of CKD and haemodialysis poses a global health challenge, particularly in nations with widespread access to medical care (23). Therefore, early intervention to halt disease progression is important, not only to reduce CV complications but also to alleviate the associated economic burden. The present review will mainly focus on subtypes 3 and 4, associated with acute and chronic reno-cardiac syndrome, respectively.

## 2. Literature search strategy

The present narrative review aims to provide an overview of the principal experimental models used to investigate CRS. Relevant publications were identified through structured searches of the PubMed/MEDLINE (https://pubmed.ncbi.nlm.nih.gov), Web of Science (https://www.webofscience.com) and Scopus (https://www.scopus.com) databases. The search strategy targeted experimental studies describing renal injury models associated with cardiac dysfunction, as well as the pathophysiological mechanisms underlying kidney/heart interactions. Keywords and their combinations included 'cardiorenal syndrome', 'renal injury', 'acute kidney injury', 'chronic kidney disease', 'renal ischemia-reperfusion', '5/6 nephrectomy', 'unilateral ureteral obstruction', 'cisplatin nephrotoxicity', 'adenine-induced CKD', 'experimental models', 'cardiac remodelling' and 'kidney-heart axis'.

The search primarily covered studies published between 1990 and 2025, although earlier landmark publications describing the development of classical experimental models were also considered when relevant. Studies were prioritized if they, i) described well-characterized experimental models of renal injury, ii) reported cardiac structural or functional consequences of kidney dysfunction or iii) provided mechanistic insights into renal/cardiac interactions, including inflammatory, metabolic and neurohormonal pathways. Both surgical and chemical models of kidney injury were considered. Additional references were identified through manual screening of reference lists from key reviews and primary articles.

Given the narrative nature of this review, the aim was not to perform an exhaustive systematic synthesis but rather to highlight representative, mechanistically informative studies that have contributed to the current understanding of experimental CRS types 3 and 4 models and their translational relevance to human disease.

## 3. Pathophysiology of cardiorenal syndrome types 3 and 4

### Cardiorenal syndrome type 3 (CRS3)

CRS3 is triggered by acute renal damage, resulting in an acute cardiac dysfunction. A central pathophysiological driver is neurohormonal activation. Indeed, AKI from haemodynamic or organic causes leads to impaired renal perfusion, interstitial oedema, tubular obstruction, hypoxia or endothelial dysfunction, and induces an activation of the renin-angiotensin-aldosterone system (RAAS) and the sympathetic nervous system (SNS), leading to pre-renal vasoconstriction, sodium and fluid retention. An increase in peripheral and systemic vascular resistance elevates cardiac afterload and workload, thereby predisposing the heart to failure (24,25). At the same time, oxidative stress and inflammation carry out a pivotal role, as AKI stimulates the release of reactive oxygen species (ROS) and pro-inflammatory cytokines, thereby directly promoting damage to endothelial and myofibrillar cells and further increasing vascular permeability (26).

Electrolyte and acid-base imbalances are also involved in CRS3 pathophysiology. When AKI is associated with tubular renal dysfunction, potassium excretion and acid-base buffering homeostasis are dysregulated, causing dyskalemia and metabolic acidosis, both influencing cardiac contractility and electrical conduction, increasing the risk of arrhythmias and sudden cardiac arrest (26,27). As the kidneys carry out a central role in regulating fluid homeostasis, AKI can result in venous congestion, increased cardiac preload and subsequent cardiac dysfunction. These alterations contribute to the development of pulmonary oedema, all of which can substantially compromise cardiac output (28). Emerging studies have also highlighted the contribution of mitochondrial dysfunction in CRS3, demonstrating that ischemic kidney injury promotes toxin accumulation, which impairs mitochondrial activity in cardiomyocytes, reducing energy production and increasing susceptibility to apoptosis (29-31). Accordingly, during AKI, excretion of uremic toxins such as indoxyl sulphate and p-cresyl sulphate is associated with direct cardiotoxic effects, worsening myocardial structure and function (32).

### Cardiorenal syndrome type 4 (CRS4)

CRS4 is a chronic process where CKD gradually worsens CV disease and increases mortality (33). Similar to CRS3, neurohormonal activation is essential since constant RAAS and SNS stimulation causes systemic hypertension, left ventricular hypertrophy (LVH) and myocardial fibrosis. Chronic renal injury leads to impaired tubulo-glomerular function, resulting in elevated plasma urea levels and accumulation of uremic toxins, particularly in advanced stages of CKD. This contributes to the development of uremic cardiomyopathy, a condition characterized by left ventricular impairment, diastolic abnormalities and pericardial effusion. Prolonged exposure to uremic toxins promotes myocardial fibrosis and microvascular damage, which are hallmarks of CRS4 (34-36).

Further aggravating CV health, CKD disrupts calcium and phosphate metabolism through altered parathyroid hormone, and vitamin D regulation. This imbalance promotes vascular calcification, arterial stiffness and endothelial dysfunction, enhancing the risk of developing ischemic heart disease (37). Another main process in CRS4 is chronic inflammation, which contributes substantially to CV complications. Indeed, this chronic inflammation promotes endothelial dysfunction, accelerates the progression of atherosclerosis and triggers adverse myocardial remodelling (38,39). These processes collectively impair vascular integrity and cardiac function, increasing the risk of ischemic events, arrhythmias and HF (40,41).

## 4. Diagnosis and evaluation

Since CRS represents a primarily pathophysiological syndrome, its clinical diagnosis is complicated by the convergence and interaction of multiple overlapping signs and symptoms. Thus, comprehensive diagnostics are necessary to determine both the nature and severity of cardiorenal involvement and to guide patient management. Clinical examination should integrate signs of cardiac and renal dysfunction, including manifestations of volume overload (for example, peripheral edema, pulmonary crackles, jugular venous distention) alongside careful monitoring of urine output. Laboratory investigations typically include the measurement of renal function, such as serum creatinine and eGFR, as well as cardiac biomarkers, including troponins and natriuretic peptides [B natriuretic peptide (BNP) or NT-proBNP], which reflect myocardial injury and wall stress. Additional parameters, including urinalysis, serum electrolyte and a complete blood count, provide insight into systemic homeostasis.

Importantly, aligning preclinical readouts with clinical staging frameworks requires not only the selection of analogous parameters but also their consistent and standardized reporting. To reduce study heterogeneity and enhance comparability with human CRS definitions, the present review recommended that preclinical investigations include a defined core outcome set covering both organ systems. For renal injury, these outcomes should include: i) Functional markers [serum creatinine, blood urea nitrogen (BUN), urine output], ii) structural damage assessments [such as kidney injury molecule-1 (KIM-1) for tubular injury] and iii) validated injury biomarkers [such as neutrophil gelatinase-associated lipocalin (NGAL) or tissue inhibitor of metalloproteinase-2 (TIMP-2) and insulin-like growth factor-binding protein-7 (IGFBP7)] where available. For cardiac dysfunction, the core dataset should include: i) Functional parameters (ejection fraction, fractional shortening), ii) structural remodelling indices (hypertrophy and/or fibrosis) and iii) circulating cardiac biomarkers (for example, BNP or NT-proBNP) when technically feasible. Explicit reporting of these parameters would facilitate alignment with clinical CRS staging, enable cross-study comparisons and strengthen translational interpretability of experimental findings.

Finally, given the bidirectional and progressive nature of cardiac and renal dysfunction in CRS types 3 and 4, early identification and intervention depend on integrated, longitudinal assessment across these parameters. A standardized and clinically anchored evaluation framework in preclinical models is essential to improving mechanistic insight and advancing therapeutic development.

## 5. Models of renal disease: Causing cardiac dysfunction

Both surgical and chemical models are widely used to study kidney diseases associated with cardiac injury. Surgical approaches, such as nephrectomy, ureteral obstruction or ischaemia, allow precise control over the location and extent of renal damage, facilitating the investigation of pathophysiological mechanisms and therapeutic interventions (42,43). By contrast, chemical models using nephrotoxic agents such as cisplatin provide a less invasive, cost-effective and scalable alternative to induce renal injury. While each method has its advantages and limitations, their combined use offers complementary insights into the cross-talk between kidneys and the heart and supports the development of targeted therapeutic strategies.

## 6. Surgical models

For >5 decades, surgical models of kidney damage have been created to mimic the pathophysiology of several renal disease causes (44-46). Among these models, 5/6 nephrectomy (Nx_5/6_) was considered to closely mimic the pathophysiology of CKD (47), as the reduction in renal mass triggers a progressive decline in kidney function. Another widely used experimental model is unilateral ureteral obstruction (UUO), which replicates key characteristics of obstructive nephropathy, a condition affecting 10% of patients with CKD (48). Finally, the renal ischaemia-reperfusion (rIR) model, which covers situations highlighting a short-term kidney damage followed by a later restoration of renal function, is frequently used to investigate AKI (49,50).

### Nx_5/6_

The Nx_5/6_ experimental model involves two separate surgical procedures to reduce renal mass, usually performed one week apart. In general, first, a total nephrectomy of one kidney is carried out and second, the poles of the contralateral kidney are excised. Pole excision varies depending on the species and the specific model used. In mice, direct ligation or excision of the renal poles is currently performed, whereas in rats, ligation of the lateral renal arteries is often used (47,51).

The Nx_5/6_ model is used to induce CKD in rodents and is employed to investigate cardiac damage secondary to kidney injury. The surgical excision of one kidney and partial infarction of the other greatly lowers functional nephron mass and reduces eGFR. It is a useful tool for studying the pathophysiology of CRS since it closely mirrors progressive renal failure and associated CV complications observed in human CKD. Reduction in nephron mass results in compensatory hyperfiltration, thereby promoting glomerular hypertension, proteinuria and further renal damage (52). This series of events includes glomerulosclerosis, tubulointerstitial fibrosis and progressive renal failure.

Multiple studies using this model have reported varying degrees of cardiac damage, occurring as early as 5 days up to 32 weeks after surgery. The CV phenotype following Nx_5/6_ includes hypertension, LVH and cardiac fibrosis. Additional characteristics, such as reduction of ejection fraction and fractional shortening, have been reported. However, according to the literature, variations in these parameters may be influenced by multiple factors, including the duration of the model, species, strain and housing conditions [Table II (53-111)]. Aside from the well-characterized renal damage, including immune cell infiltration (53-55,63), elevated levels of pro-inflammatory cytokines and chemokines (IL-6, TNF-α, IL-1β and monocyte chemoattractant protein-1) (56,60,64), and increased interstitial and glomerular fibrosis (53,61,63,65), this model also exhibits a notable increase in apoptosis markers within renal and cardiac tissue (57,60,66).

The Nx_5/6_ model promotes structural and functional cardiac alterations, as evidenced by increased levels of markers such as atrial natriuretic peptide (ANP) and BNP, which begin to rise ~5 weeks after surgery and remain persistently elevated in both cardiac and plasma over extended periods (58,59,62,67,68). Similar to the kidney, the heart shows early immune cell infiltration, including T cells and monocytes, as early as day 5 following injury (112). Along with fibrotic markers such as fibronectin, collagens I, III, IV and V, TGF-β, and α-smooth muscle actin (α-SMA) (53-55,69-74), pro-inflammatory cytokine expression highly increases (53,60,69,73). This is followed by an increase in apoptotic cells 4 weeks after surgery (58,66,74-76,79). This model also exhibits endothelial damage beyond the heart and kidneys, as demonstrated by increased expression of vascular adhesion molecules and elevated levels of vasoconstrictors such as endothelin-1 and norepinephrine (69,81,82,113). These molecular, structural and vascular alterations aggravate the course of CRS by promoting increased arterial stiffness and impaired vasomodulation, thereby limiting renal adaptation to haemodynamic fluctuations.

Systemic complications following haemodynamic and inflammatory alterations may promote an elevated oxidative stress in both renal and cardiac tissues over time (54,69,71,76-78,80), following heightened protein (79) and lipid oxidation (76,80). This seems to be partly due to a decrease in erythropoietin production after Nx_5/6_ (60,89), decreasing both haemoglobin and haematocrit levels (60,66,76,83), thus maintaining an oxidative condition. In addition, haemodynamic alteration is maintained due to SNS activity modulation (84-86) and activation of the RAAS (62,68,80,84,87,88,90,114). Experimental therapies targeting antioxidant delivery and pharmacological RAAS blockade have been reported, showing varying levels of renal and cardiac protection (115,116). Moreover, Nx_5/6_ exhibited metabolic disturbances, including raised ionic and lipid metabolite blood levels (68,69,71,91-94), despite increased diuresis in affected animals (61,93). These findings highlighted the possibility of dietary approaches to help slow the disease progression. To resume, the Nx_5/6_ model is a well-established method for inducing CKD and studying its systemic effects, particularly CRS. By substantially reducing nephron mass, it mimics key features of human CKD, including inflammation, fibrosis, oxidative stress and CV complications such as hypertrophy and fibrosis.

### UUO

The UUO model involves surgical obstruction of one ureter, either partially or completely. This is a well-established model that causes gradual renal damage marked by tubulointerstitial inflammation and later fibrosis. Ureteral occlusion increases intratubular pressure, initiating nephron damage. This mechanical stress, along with exposure to harmful substances, induces tubular cell damage, triggering aberrant cellular activation, dysregulated cell cycle progression or cell death (117). Notably, this model allows researchers to investigate tissue damage without renal dysfunction (defined by a decrease in eGFR or an increased serum creatinine); thus, cardiac complications such as LVH and cardiac fibrosis still manifest [Table III (98,112,118-136)].

The UUO model is characterized by the local production of cytokines, chemokines and adhesion molecules, thus promoting the recruitment of immune cells. Several studies have shown that neutrophil and macrophage infiltration occurs ≤3 days following ureteral obstruction (137-139), whereas CD4^+^ and CD8^+^ lymphocyte infiltration was more pronounced after 7 days (140-142). All these elements aggravate tubule cell damage by intensifying inflammation.

UUO causes extensive functional and structural alterations in the kidney, including tubular atrophy, interstitial fibrosis and glomerular sclerosis beyond inflammation. Activation of the TGF-β/Smad signalling pathway, upregulation of pro-fibrotic mediators such as connective tissue growth factor and plasminogen activator inhibitor-1 (143,144), drives these pathological changes. This promotes in particular myofibroblast activation, expressing α-SMA and further, the progression of interstitial fibrosis characterized by an increase in activated fibroblasts and the accumulation of extracellular matrix components (118,122,123).

Beyond its renal effects, UUO also exerts a considerable influence on the CV system, contributing to cardiac remodelling and dysfunction through systemic inflammation, oxidative stress and altered haemodynamics. Indeed, even before the onset of overt kidney failure, early signs of cardiac hypertrophy and fibrosis suggest that renal injury can directly contribute to cardiac remodelling. Increased oxidative stress, upregulation of the TGF-β/Smad signalling pathway and elevated inflammatory cytokine expression are associated with the fibrotic modifications in the heart (119,123,124). In addition to myocardial fibrosis, UUO may promote endothelial dysfunction, increased arterial stiffness, poor vasodilation and elevated cardiac damage markers such as BNP and ANP (119,145,146), therefore aggravating CRS. The extent of these CV alterations varies depending on the rodent strain and experimental conditions.

Further aggravating the renal injury, the RAAS is upregulated as it drives pre-renal vasoconstriction, sodium retention and inflammation (122,147). Experimental studies using the UUO model have shown that inhibition of the RAAS by angiotensin-converting enzyme (ACE) inhibitors or aldosterone antagonists may reduce cardiac fibrosis, hypertrophy and vascular dysfunction (119,122,124,125). These results highlight the therapeutic potential of RAAS blockade in treating CKD-associated CV disease.

In conclusion, along with notable CV effects including cardiac hypertrophy and fibrosis, the UUO model appropriately shows the progression of renal injury marked by tubulointerstitial inflammation and later fibrosis. It is a useful instrument for investigating cardiorenal interactions and possible therapies since it highlights the essential roles of inflammation, TGF-β/Smad signalling and RAAS activation in driving both renal and cardiac pathology.

### rIR

rIR injury represents a key model for studying AKI and transition to CKD. This model could also contribute to the knowledge of CRS, a condition in which AKI promotes cardiac dysfunction and structural damage. Reperfusion, after renal ischemia induced by a transient renal pedicle clamping, which causes a brief period of reduced blood flow, results in extensive cellular injury due to the abrupt restoration of oxygen and nutrients. Moreover, this process leads to oxidative stress, inflammation and tissue damage. The rIR model can be applied in different ways, depending on the type of ischemia (unilateral or bilateral), the duration of ischemia or the need to increase the renal severity of the model through unilateral nephrectomy, promoting transient renal dysfunction, regardless of the reperfusion time (Table III). The pathophysiology of rIR may lead to mitochondrial dysfunction, enhanced ROS generation and activation of pro-inflammatory pathways (148).

Systemic release of plasma pro-inflammatory cytokines (TNF-α, IFN-γ, IL-1β, IL-6 and IL-10) is one of the major effects of rIR (126-128). These cytokines stimulate NF-κB and JAK/STAT pathways, contributing to cardiac inflammation and fibrosis (128,149,150). Endothelial dysfunction, which is characterized by impaired vasodilation and increased vascular permeability (151), also results from the inflammatory response, thus contributing to renal and cardiac dysfunction. Oxidative stress is also observed after rIR. ROS accumulation results in lipid peroxidation, protein oxidation and mitochondrial damage, and can impair cardiac contractility and induce apoptosis in cardiomyocytes (127,129,152). Additionally, oxidative stress could induce the activation of RAAS (153), promoting hypertension, cardiac hypertrophy and myocardial fibrosis.

rIR can also alter cardiac function, inducing cardiomyocyte calcium handling by cardiomyocytes. Perturbation of intracellular calcium homeostasis results in the inability of the cell to regulate excitation-contraction coupling, predisposing to arrhythmias and decreasing cardiac output (128,154). Furthermore, the renal dysfunction leads to the accumulation of uremic toxins such as p-cresyl sulphate and indoxyl sulphate which also contribute to cardiomyocyte injury, increased oxidative stress and myocardial fibrosis (155,156).

Neurohormonal activation secondary to rIR is characterized by elevated SNS activity and impairment in the RAAS (157,158). The resulting alterations cause a dysregulation of the vasoreactivity, and the heart afterload promotes left ventricle hypertrophy (159). Fluid retention from chronic volume overload adds to the worsening of cardiac remodelling and the risk for HF.

Pharmacologic interventions that reduce inflammation, oxidative stress and neurohumoral activation have been shown to attenuate heart injury in experimental models of rIR. Indeed, antioxidant, anti-inflammatory and RAAS-inhibiting drugs (including ACE inhibitors or angiotensin receptor blockers) can decrease myocardial fibrosis and enhance cardiac function after rIR (127,130,160-162). For instance, pharmacological inhibition of the lectin Galectin-3 prevented cardiac injury following AKI, by reducing renal damage and inflammation, thereby limiting cytokine release, cardiac macrophage infiltration and fibrosis, ultimately restoring cardiac function (126).

Hence, rIR injury may emerge as a major element of CRS, connecting AKI with secondary cardiac dysfunction through systemic inflammation, oxidative stress, calcium mishandling, neurohumoral activation and uremic toxicity. Elucidating these mechanisms is key to the development of therapeutic approaches targeted at blocking the advancement of CRS3 and improving patient outcomes.

## 7. Chemical models

Among the various chemical nephrotoxic agents used in experimental nephrology, cisplatin and adenine stand out as the most widely used compounds upon which to model AKI and CKD, respectively. These nephrotoxins induce reproducible renal injury through well-defined mechanisms; cisplatin causes acute tubular necrosis and inflammation, while adenine leads to tubulointerstitial fibrosis and progressive CKD. Both models have been useful in elucidating the systemic consequences of kidney dysfunction on the heart, enabling the investigation of haemodynamic alterations, oxidative stress, fibrosis and inflammatory pathways that contribute to cardiac remodelling in cardiorenal syndromes [Table IV (98,163-187)].

### Cisplatin

Cisplatin is a widely used chemotherapeutic agent whose major dose-limiting side effect is acute nephrotoxicity, primarily targeting the proximal tubules. In rodent models, systemic administration induces a marked decline of renal function, proximal tubular necrosis (188), inflammation (189) and oxidative stress (190), closely reproducing the clinical profile of nephrotoxic AKI observed in patients with cancer (163,191-193). The severity of injury depends on the dose, strain and treatment duration, and in prolonged regimens, may progress to persistent injury with interstitial fibrosis.

Although this model is primarily used to study drug-induced nephrotoxicity, multiple studies have shown that cisplatin induced AKI. In rodent models, cisplatin-induced AKI is typically induced using doses ranging from 3 to 10 mg/kg, mostly administered intraperitoneally, either as a single injection or as repeated weekly injections over 1-3 weeks, depending on the desired severity of renal injury. These regimens produce dose-dependent tubular injury, inflammation and oxidative stress (Table IV). In C57BL/6 mice, weekly regimens of 6 mg/kg for 3 weeks may reduce ejection fraction and stroke volume, induce LVH, impair diastolic relaxation and promote myocardial fibrosis (163). These changes are accompanied by cardiomyocyte apoptosis, activation of inflammatory pathways and dysregulation of PI3K/Akt signalling (164,165). Other studies have identified endoplasmic reticulum stress and mitochondrial ultrastructural damage as central drivers of contractile impairment (166) as well as gut microbiota dysbiosis which exacerbates systemic inflammation (163). Antioxidant and anti-inflammatory interventions have demonstrated both renal and cardiac protection. Taurine (165), salvianolic acid B (167), maltol (164), probiotics (*Lactobacillus*) (163) and polyphenol-rich plant extracts (168) may reduce oxidative stress, attenuate myocardial fibrosis and preserve left ventricular function.

Taken together, the cisplatin-induced AKI model provides a reproducible and clinically relevant platform for studying kidney and heart interactions in CRS3, particularly during the acute phase. However, its application to the study of chronic cardiac remodelling and dysfunction is more limited, as the majority of protocols focus on short-term outcomes and CV injury is typically secondary to renal and systemic toxicity. Extended or repeated dosing regimens may help to model the AKI to CKD transition and to characterize mechanisms of sustained cardiac injury.

### Adenine

Adenine-induced CKD is a well-established, non-surgical rodent model extensively used to study CRS4, in which chronic renal injury contributes to progressive CV disease. This model is typically generated through dietary administration of adenine at concentrations ranging from 0.15 to 0.75%, incorporated into rodent chow. Protocol duration generally varies from 2 to 20 weeks, depending on the severity of renal injury required. Alternative protocols include gavage or intraperitoneal administration, although dietary exposure remains the most widely used approach (Table IV). The adenine is metabolized in the liver to 2,8-dihydroxyadenine, a poorly soluble metabolite that precipitates in renal tubules. This crystal deposition induces tubular obstruction, inflammation and progressive tubulointerstitial fibrosis, ultimately leading to a sustained decline in renal function (194). This model faithfully reproduces several hallmarks of human CKD, including elevated serum creatinine and BUN, azotaemia, proteinuria, altered urine output, mineral metabolism disorders, systemic inflammation and interstitial fibrosis (171,187).

Importantly, multiple studies have demonstrated that adenine-induced CKD is associated with consistent CV alterations characteristic of CRS4 (172,173). For instance, prolonged administration of adenine (0.15% for 20 weeks) has been shown to impair systolic function (reduced ejection fraction) and induce myocardial fibrosis with extracellular matrix accumulation (174). Early metabolic remodelling has also been described, with fibroblast growth factor 23 (FGF23)-FGFR4 signalling driving mitochondrial dysfunction and concentric LVH in C57BL/6 mice (175). Other studies report diastolic dysfunction with preserved systolic performance, mimicking the HF with the preserved ejection fraction (HFpEF) phenotype frequently observed in patients with CKD (176). Additional haemodynamic changes include hypertension, altered circadian blood pressure rhythms and non-dipping profiles contributing to CV burden (177-179,187).

Additionally, sex-specific differences have been documented. For instance, in Wistar rats, both sexes developed myocardial fibrosis under adenine feeding, but males exhibited more severe renal impairment, concentric hypertrophy and alterations in ERK1/2 and oestrogen receptor signalling pathways (178). Furthermore, adenine-induced uraemia increases myocardial susceptibility to secondary insults; ischaemia-reperfusion injury severity is exacerbated in uremic rats, particularly under air pollution exposure, underscoring heightened mitochondrial vulnerability and oxidative stress (186).

Collectively, these findings establish the adenine model as a robust and mechanistically informative platform for studying CRS4. It recapitulates key features of renal injury, systemic inflammation, myocardial fibrosis, cardiac hypertrophy and haemodynamic alterations relevant to human disease. Nevertheless, certain limitations must be considered, including dose-dependent weight loss, variability in disease severity based on dietary concentration and duration and limited progression to glomerulosclerosis (194). Despite this, adenine-induced CKD remains a cornerstone experimental model for dissecting the mechanisms linking chronic renal dysfunction to adverse CV remodelling.

Overall, both surgical and chemical models provide complementary and well-established platforms for studying renal disease-induced cardiac dysfunction in CRS (Table V). Surgical approaches such as Nx_5/6_, UUO and rIR enable controlled investigation of CKD and AKI-driven mechanisms, including inflammation, fibrosis, oxidative stress and neurohumoral activation, all of which contribute to cardiac remodeling and dysfunction. In parallel, chemical models such as cisplatin and adenine offer reproducible and scalable alternatives that recapitulate acute and chronic renal injury and their systemic cardiovascular consequences. While each model captures specific aspects of CRS pathophysiology, none fully reflects the complexity of human disease. Therefore, their combined and context-dependent use remains essential for elucidating kidney-heart crosstalk and advancing translational therapeutic strategies.

## 8. Clinical applicability (renal disease with cardiac consequences)

Animal models are indispensable for investigating the complex pathophysiology of CRS, offering controlled and reproducible environments that are difficult to achieve in human studies enabling detailed exploration of disease mechanisms and therapeutic interventions (195,196). A major advantage of these models lies in the ability to tightly regulate genetic, dietary and environmental variables, thereby enhancing experimental reproducibility and facilitating the validation of mechanistic hypotheses and the preclinical testing of novel therapies (195-198). In addition, animal models allow investigation of organ crosstalk and the progression of dysfunction in one organ following injury to the other, processes that are difficult to isolate in clinical settings (197-199). The relatively rapid progression of the disease in these models, occurring over weeks or months rather than the years seen in humans, facilitates efficient study of disease onset, trajectory and treatment response (196,200,201). Consequently, a range of CRS phenotypes, including those driven by CKD, HF or metabolic syndrome, can be simulated to support the development of targeted interventions (196,201,202).

However, translating findings from experimental models to clinical CRS remains challenging. In clinical practice, the interpretation of renal and cardiac biomarkers in CRS is often complicated by several confounding factors. Baseline CKD may alter serum creatinine and natriuretic peptides levels (203,204), while fluid overload, haemodynamic instability or diuretic therapy can affect urine output and circulating biomarkers (1,205,206). Similarly, medications such as vasopressors, renin-angiotensin system inhibitors or nephrotoxic drugs may modify both renal and cardiac function (207-209). These factors should be considered when interpreting preclinical findings, particularly in CRS types 3 and 4, where establishing the temporal relationship between renal and cardiac dysfunction may be difficult.

**Figure 1:**
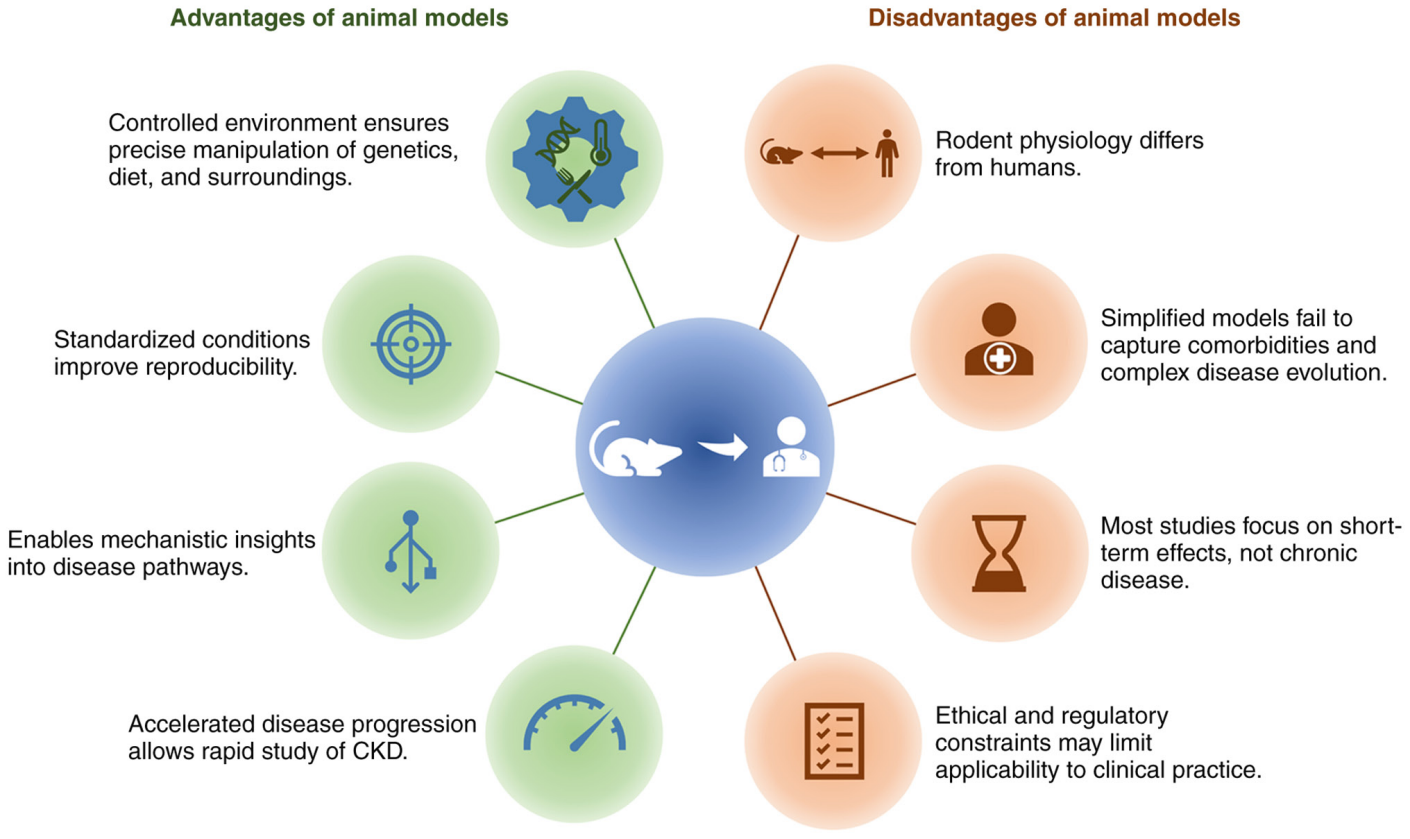
Schematic illustration of the translational relevance of animal models in renal-cardiac research, highlighting principal advantages and disadvantages compared with human disease. CKD, chronic kidney disease.

Despite their strengths, current animal models have several limitations. Species-specific physiological differences, particularly in cardiovascular and renal systems, may limit the extrapolation to humans (195,199,210). Moreover, the majority of models focus on isolated organ injury or acute pathological processes and therefore fail to capture the chronic, multifactorial and progressive nature of human CRS (197,198,211). In particular, the frequent absence of common comorbidities such as diabetes, hypertension, atherosclerosis and ageing represents a major gap as these factors substantially shape disease trajectory and patient outcomes (196,200). To improve clinical fidelity, experimental models can be refined by combining classical renal injury models with established cardiometabolic conditions. For example, renal ischaemia-reperfusion or adenine-induced CKD may be studied using a diabetic background (for example, streptozotocin-induced diabetes or db/db mice), hypertensive models (for example, angiotensin II infusion or spontaneously hypertensive rats) or atherosclerosis-prone strains such as ApoE^−^/^−^ mice. The incorporation of aged animals or dietary interventions, including high-fat or high-phosphate diets, can further approximate the metabolic and vascular environment typical observed in human CRS. Importantly, incorporating comorbidities enhances clinical fidelity but complicates interpretation, as they alter renal baselines, biomarkers and cardiac remodelling. Combined models improve translational relevance but increase complexity, so model choice should align with mechanistic vs. translational study goals. Ethical and regulatory constraints, particularly large animal studies, must also be considered (198,210) (Fig. 1) and the clinical relevance of selected preclinical models are discussed [Table VI (51,212-220)].

### Nx_5/6_ model

As aforementioned, the Nx_5/6_ model is one of the most widely used experimental systems to study CKD and its CV complications, making it particularly relevant to the study of CRS4/3. The model reliably reproduces cardiac complications commonly seen in advanced CKD. From a translational perspective, this model provides a clinically relevant platform to investigate mechanisms and test interventions targeting the CV sequelae of CKD.

The cardiotoxic effect of uraemia has been evaluated through this model, in the cardiac pathology of rats with Nx_5/6_ (without acute myocardial infarction), where capillary/myocardial cell mismatch and interstitial fibrosis were found. Similarly, autopsy studies have shown that the number of capillaries per myocardial cell decreases, and fibrosis increases in uremic patients (221,222). Accumulation of uremic toxins not only affects myocardial remodelling but is also associated with an increase in the incidence of ischemic heart disease. Indeed, Nx_5/6_ induced uremic rats have lower myocardial cell volume density, a substantially larger ratio of infarct area and reduced intrinsic tolerance of myocardium to ischemic injury (223,224). These findings suggest that uremic cardiomyopathy may increase susceptibility to ischemic injury, supporting clinical observations that patients with advanced CKD have a higher risk of cardiovascular events. They also advocate for the immediate use of currently available anti-remodelling strategies, such as β-blockers, ACE inhibitors or angiotensin II type 1 (AT_1_) receptor blockers, in patients with CKD after an acute myocardial infarction.

The nephron loss induced by Nx_5/6_ leads to chronic hypertension due to the alteration of sodium and water excretion, which impairs cardiac contractility and diastolic strain, while increasing cardiac mass. The reduction in cardiac contractility is largely attributed to LVH and diastolic dysfunction, characterized by increased myocardial cell diameter and volume, along with a decreased capillary density, as observed in experimental models of renal failure.

The Nx_5/6_ model has also contributed to an understanding of the systemic pathways increasingly recognized in clinical settings, such as the gut-kidney-heart axis. Alterations in gut microbiota and microbial metabolites in Nx_5/6_ animals have been associated with cardiorenal dysfunction, suggesting potential therapeutic targets with emerging clinical interest (225). Despite the aforementioned strengths, the clinical translatability of the Nx_5/6_ model is limited by several factors. It does not fully replicate the comorbid landscape of human CKD, notably lacking common features such as diabetes, obesity and atherosclerosis, which often exacerbate CV risk. Moreover, differences in CV responses across rodent strains introduce variability and may complicate the extrapolation to diverse human populations (90).

### UUO-a model of obstructive nephropathy and renal fibrosis

From a translational standpoint, UUO has been highly involved in transcriptomic profiling studies, revealing both coding and non-coding RNA signatures that may serve as biomarkers or therapeutic targets for renal fibrosis and potentially CRS (226). Furthermore, due to its consistency and rapid progression, the model is well-suited for early-phase drug screening and mechanistic validation of nephroprotective compounds.

The UUO model is a key experimental tool for studying obstructive nephropathy, hydronephrosis and renal interstitial fibrosis. It induces rapid urine obstruction by ligating one ureter, resulting in hydronephrosis and renal oedema. Clinically, hydronephrosis is characterized by loss of renal medullary tissue, most often as a consequence of obstructive nephropathy. In humans, the leading causes include congenital anomalies, urolithiasis, malignancy or fibrotic inflammatory processes. Both paediatric and adult case reports have shown an association between hydronephrosis and elevated blood pressure, where the surgical relief of the obstruction often alleviates the hypertension (227,228). In a study using Sprague Dawley rats with spontaneous hydronephrosis, impaired cardiac autonomic regulation, including elevated resting heart rate, reduced heart rate variability and blunted baroreflex sensitivity, has been reported. These changes occurred independently of peripheral RAAS activation and were attributed to enhanced angiotensin II activity in the nucleus tractus solitarius, a key point in clinical CRS (229).

However, the UUO model does not fully capture the chronic, recurrent and multifactorial characteristics of human CKD and obstructive uropathy. Most importantly, UUO induces profound structural changes with relatively modest or absent long-term functional decline in the contralateral kidney, which limits its utility in modelling the progressive renal impairment characteristic of CRS (230). Despite these constraints, emerging evidence suggests that prolonged UUO can lead to systemic consequences, including cardiac fibrosis, inflammation and lymphangiogenesis, indicating its partial utility in modelling the renal-to-cardiac axis central to CRS 3/4 (214,231). Species-specific signalling must be considered when using animal models. Although endothelial-to-mesenchymal transition may play a role in fibrosis in UUO mice, its contribution seems limited in human kidneys (232,233).

### rIR model

rIR injury models are widely employed in preclinical research to simulate AKI, delayed graft function and early post-transplant complications. These models are also highly relevant for studying CRS, particularly type 3, in which acute renal insults lead to secondary cardiac dysfunction, and type 4, where persistent renal injury contributes to progressive CV remodelling. rIR models replicate key pathophysiological events observed in human AKI and kidney transplantation. From a translational perspective, these models recapitulate key features of human ischemic injury and post-transplant pathology, including the transition from acute to chronic injury characterized by interstitial fibrosis, tubular atrophy and persistent inflammation. In clinical settings involving rIR, such as kidney transplantation, partial nephrectomy, renal artery angioplasty, cardiopulmonary bypass, aortic bypass surgery or other medical conditions, these procedures remain among the most frequent causes of acute renal failure.

Molecular and transcriptomic signatures from these models show substantial overlap with human kidney transplant biopsies, supporting their utility in preclinical evaluation of targeted therapies and identification of prognostic biomarkers (234). Some biomarkers validated by animal experiments have also been applied to predict human AKI, including neutrophil NGAL, liver-type fatty acid-binding protein (L-FABP), KIM-1, tissue inhibitor of metalloproteinase-2 (TIMP-2) and insulin-like growth factor-binding protein-7 (IGFBP7). Serum IL-6 and IL-8 have been confirmed as early indicators of AKI in patients undergoing cardiac bypass surgery (235-237). Importantly, these models have also revealed the systemic impact of renal ischemia on distant organs, including the heart. Experimental data show that cytokine release following rIR can contribute to myocardial inflammation and dysfunction, offering mechanistic insight into the renal-to-cardiac axis that defines CRS (238-240). As such, these models are clinically relevant for investigating the early inflammation and haemodynamic drivers of cardiac injury following AKI.

The rIR rodent model shares similarities with certain aspects of AKI in humans, such as kidney tissue damage, tubular epithelial cell proliferation, inflammatory response and fibrosis. In both rats and mice, ischemic injury leads to the proliferation of proximal renal tubular cells (241). Similar evidence of post-injury recovery response has also been observed in human biopsy samples after ischaemic or renal injury, as well as in cases of delayed transplant function (242).

However, the regenerative capacity of human kidneys is limited, resulting in a slower and incomplete recovery process after AKI compared with that of mice (242,243). Therefore, the progression and severity of diseases in rodent rIR models may not be the same as in human AKI. Renal tubular injury is evident in human kidneys, but necrosis after ischemia appears patchy, while in rodent models, it is more pronounced as cell death. Furthermore, these models lack key human-specific modifiers such as alloimmune responses, chronic immunosuppression and patient-level comorbidities (for example, diabetes or CV disease), which influence both renal and cardiac outcomes in clinical settings (216).

### Cisplatin

Cisplatin is a widely used chemotherapeutic agent with well-documented nephrotoxicity and emerging evidence of CV complications in humans. In preclinical research, cisplatin-induced AKI serves as a clinically relevant model for investigating the kidney-heart axis in patients with cancer, particularly within the framework of CRS3, where AKI precipitates or exacerbates cardiac dysfunction. Cisplatin-induced AKI may contribute to systemic endothelial dysfunction, electrolyte disturbances and a pro-thrombotic state, factors known to impact CV homeostasis. When compounded by high phosphate diets, aging or Klotho deficiency, these models exhibit uremic vasculopathy and cardiac remodelling, reinforcing their utility for studying CRS (244,245). Importantly, combination models that integrate cisplatin exposure with dietary or genetic risk factors (for example, high phosphate, aged mice, Klotho deficiency) allow the simulation of AKI-to-CKD progression and concurrent CV injury, thereby extending the model's relevance to CRS 3/4 (244,245). The mechanistic pathways activated in cisplatin-induced injury may not fully represent those in metabolic or haemodynamic CKD. Additionally, preclinical models often lack confounding factors common in patients with cancer, such as polypharmacy, pre-existing CV disease and heterogeneous tumour biology. The clinical relevance of these models is underscored by known risk factors for cisplatin nephrotoxicity, including pre-existing CKD, CV disease and NSAID use (246). Standard preventive strategies, such as intravenous hydration, magnesium supplementation and mannitol-induced diuresis, are mirrored in preclinical designs. Novel therapeutic approaches, including antioxidant compounds, mitochondrial protectants and natural products, are currently under investigation in these models (247-249).

### Adenine

Adenine-induced CKD models in rodents induce tubulointerstitial injury, renal insufficiency and a spectrum of metabolic disturbances that closely mimic the human CKD phenotype. Animals consistently develop elevated plasma urea and creatinine, anaemia, hyperphosphatemia, hypocalcemia and altered levels of FGF23, all of which are hallmarks of advanced CKD and drivers of CV disease in patients (176,196,250). Cardiac alterations observed in adenine-induced CKD include LVH, increased end-diastolic pressure and diastolic dysfunction with preserved systolic function. This mirrors the clinical presentation of HFpEF, a common cardiac manifestation in patients with CKD (176,251). Furthermore, this model induces substantial cardiac oxidative stress, inflammation and DNA damage, with upregulation of Nrf2 and pro-inflammatory cytokines (250,252), but also endothelial dysfunction, characterized by impaired nitric oxide (NO)-dependent vasodilation in the aorta, and a prothrombotic state with elevated platelet counts, both of which reinforce the model's relevance for studying vascular contributions to CRS and CV risk in CKD and mimic the closely reproduce the complex CV alterations observed in human (250,253). Although the abrupt onset and severity of renal dysfunction in experimental models may differ from the more gradual and heterogeneous progression seen in clinical settings, this discrepancy may limit the generalizability of therapeutic outcomes.

## 9. Conclusion

CRS3 and CRS4 highlight the complex bidirectional interactions between renal and cardiac dysfunction through shared mechanisms including inflammation, oxidative stress and neurohormonal activation. Experimental models such as Nx_5/6_, UUO, rIR, cisplatin and adenine-induced injury reproduce key aspects of these syndromes and have substantially improved the mechanistic understanding of kidney-heart interactions. However, important limitations remain, including species-specific differences, the frequent absence of common comorbidities, and the limited ability to reproduce the progressive and multifactorial nature of human CRS. Addressing these limitations, future research should prioritize the development of models incorporating cardiometabolic comorbidities, improved standardization of experimental readouts aligned with clinical CRS definitions and integrative multiorgan models that better capture the chronic and bidirectional progression of cardiorenal dysfunction. Such approaches may help bridge the gap between experimental discovery and clinical translation.

## Figures and Tables

**Table I:** Characteristics of different CRS subtypes.

CRS type	Primary organ dysfunction	Secondary organ injury	Timing of dysfunction	Description
Type 1	Heart	Kidney	Acute	Acute worsening of cardiac function leading to acute kidney injury.
Type 2	Heart	Kidney	Chronic	Chronic cardiac dysfunction leading to progressive chronic kidney disease.
Type 3	Kidney	Heart	Acute	Acute kidney injury leading to acute cardiac dysfunction.
Type 4	Kidney	Heart	Chronic	Chronic kidney disease contributes to chronic cardiac dysfunction.
Type 5	Systemic condition	Heart and kidney (simultaneous)	Acute or chronic	Systemic disorders (for example, sepsis and diabetes) cause simultaneous cardiac and renal injury.

**Table II:** Functional and structural parameters in Nx_5/6_ surgical rodent models.

Strain	Duration	Renal function			Renal structure		Cardiac function			Cardiac structure		(Refs.)
		Cr	BUN	UP	Fib	Tub/glm	BP	EF	FS	LVH	Fib	
B6	5.0 d	η	η	η	η	η					Infil.	(53)
B6	2.0 w		η	η	η	η G.A.	η					(95)
B6	4.0 w	η	η	η	η		-	-	-	-	η	(53,56,75,96)
B6	6.0 w	η	η				-			-		(97)
B6	8.0 w	η	η	η	η		-	-	-	η	η	(57,72,98,99)
B6	8.0 w	η	η					τ	τ			(100)
B6	10.0 w	η	η		η	η G.A.	η	-	-	η	η	(58)
B6	12.0 w	η	η	η	η	η	η	τ	τ	η	η	(73,74,76,89,90)
B6	16.0 w	η	η				η	τ	τ	η	η	(62)
Wis	10.0 d	-	η				η				η	(101)
Wis	2.0 w	η	η	η			η			-		(77,91)
Wis	4.0 w	η	η	η	η	ME	η	τ	τ	η	η	(70,82,83,91)
SD	4.0 w	η	η	η			η	η		η	η	(69,84)
SD	5.0 w	η	η	η		necr	η	-		η	η	(59,92)
SD	5.5 w	η					η			η		(102)
Wis	6.0 w	η	η	η			η			η		(80,103)
SD	6.0 w	η	η	η	η	η	η		-	-	η	(66)
SD	7.0 w	η	η	η	-		η	-		η	-	(65,104)
Wis	8.0 w	η	η	η	η		η	-			η	(64,105)
SD	8.0 w	η	η	η			η	η	η	η	η	(71,78,85)
SD	8.0 w	η	η	η			-			η		(68,93)
SD	8.0 w	η	η	η	η		η	τ	τ	η	η	(86)
Wis	8.5 w	η	η	η			η	-	-	-		(106)
Wis	8.6 w	η	η	η	η		-					(63)
Wis	9.0 w	η	η	η			-	-		-	η	(107)
Wis	10.0 w	η	η				η					(108)
SD	10.0 w	η		η			η	-	-	η	η	(54)
Wis	11.0 w			η	η		η					(61)
SD	12.0 w	η	η	η			η			η	η	(81,88)
SD	13.0 w	η					η	τ		η		(79)
Wis	15.0 w			η			η					(109)
Wis	16.0 w	η	η		η		η			η	-	(110)
SD	16.0 w	η	η	η	η	τ N°glm, η G.A.	η	-	τ	η	η	(55,111)
Wis	18.0 w	η	η		η		η			η	-	(67)
SD	20.0 w	η	η	η			η	-		η		(87,94)
SD	24.0 w			η			η		τ			(60)

η, increase; τ,. decrease; -, no change; B6, C57BL/6; Wis, Wistar; SD, Sprague Dawley; Cr, plasma/serum creatinine; BUN, blood urea nitrogen; UP, urine protein; Fib., fibrosis; Tub/glm, tubular/glomerular damage; BP, blood pressure; EF, ejection fraction; FS, fractional shortening; LVH, left ventricular hypertrophy; necr, necrosis; G.A., glomerular area; ME, mesangial expansion; N°glm, glomerular number; Infil., infiltration of immune cells; d, day; w, week.

**Table III:** Functional and structural parameters in UUO and rIR surgical rodent models.

Strain	Model	Duration	Renal function			Renal structure		Cardiac function			Cardiac structure		(Refs.)
			Cr	BUN	UP	Fib	Tub/glm	BP	EF	FS	LVH	Fib	
B6	UUO	7-14 d	-	-		η		-					(118,120)
B6	UUO	21 d	η	η			η	η	-		η	η	(119)
B6	UUO	28 d						-	τ		η	η	(121)
B6	UUO	56 d								τ	η	η	(126)
SD	UUO	7 d						η					(131)
SD	UUO	14 d	-	η	-	η	η	-					(122)
Wis	UUO	15 d	η	η		η						η	(125)
Wis	UUO	28 d	η	-		η			-	-		η	(123)
Wis	UUO	180 d										η	(124)
B6	IR	30 min/48 h						-	τ		η	η	(121)
B6	IR	60 min/2 d	-	η					-	-	η		(128)
B6	IR	60 min/15 d									η	η	(132)
B6	IR + Nx	25 min/28 d	-	-						τ		η	(126)
B6	IR + Nx	30 min/16 w	η	η					-	-	η	η	(98)
B6	BIR	30 min/72 h	η	η						τ	-		(129)
SD	IR + Nx	30/85 min						-					(133)
Wis	IR + Nx	50/60 min						-					(134)
SD	BIR	30/180 min	-				necr	-					(135)
Wis	BIR	45 min/5 m	η		η	η		-			η	η	(112)
SD	BIR	45 min/3 h	η	η				τ	τ				(130)
SD	BIR	50 min/48 h	η	η									(136)
SD	BIR	60 min/24 h	η	η			necr	η	τ	τ		η	(127)

η, increase; τ, decrease; -, no change; B6, C57BL/6; Wis, Wistar; SD, Sprague Dawley; UUO, unilateral ureteral obstruction; IR, ischemia-reperfusion; BIR, bilateral IR; Nx, nephrectomy; Cr, plasma/serum creatinine; BUN, blood urea nitrogen; UP, urine protein; Fib., fibrosis; Tub/glm, tubular/glomerular damage; BP, blood pressure; EF, ejection fraction; FS, fractional shortening; LVH, left ventricular hypertrophy; necr, necrosis; d, day; w, week.

**Table IV:** Functional and structural parameters in chemical rodent models of renal disease.

Strain	Doses	Adm	Time	Renal function			Renal structure		Cardiac function			Cardiac structure		(Refs.)
				Cr	BUN	UP	Fib	Tub/glm	BP	EF	FS	LVH	C-Dam	
Cisplatin														
B6	3 mg/kg/d	i.p.	7 d							τ	τ			(167)
ICR	3 mg/kg/2d	i.p.	9 d										η	(164,169)
B6	6 mg/kg/w	i.p.	21 d							τ	τ	η		(163,170)
Balb/c	7 mg/kg/d	i.p.	27 d										η	(168)
B6	10 mg/kg/d	i.v.	7 d									η	η	(166)
Swiss	10 mg/kg/d	i.p.	7 d										η	(165)
Adenine														
B6	0.15%	Feed	12 w		η							-		(175)
B6N	0.15%	Feed	16 w	η	η		-		-	-	-		η	(180)
B6	0.15%	Feed	20 w	η	η		η	η					η	(174)
B6N	0.20%	Feed	2 w	η	η				-					(177)
B6	0.20%	Feed	6 w	η	η		η	η					η	(181)
B6J	0.2-0.05%	Feed	2-4 w	η	η		η		-	τ				(180)
B6	0.2-0.1-0.2-0.1-0.2%	Feed	2-2-1-2-1 w					η				-		(182)
B6	0.2-0.05-0.2%	Feed	3-3-3 w					η					η	(183)
B6	0.20%	Feed	16 w	η	η					η	η	-	η	(98)
B6	0.25%	Feed	6 w		η		η						η	(184)
Wis-SD	0.25%	Feed	8 w	η	η	η	η	η	η	-	-	η	η	(172,179)
Wis	0.25%	Feed	16 w	η	η	η	η	η	η	-	-	η	η	(173,178)
129/Sv	0.50%	Feed	13 w	η	η					-	-			(180)
SD	0.5-0.3-0.15%	Feed	3-2-8 w	η					η	-	-	η	η	(176)
SD	0.75%	Feed	3 w	η	η								η	(171)
Alb rats	0.75%	Feed	4 w	η	η	η			η					(187)
Wis	600 mg/kg/d	Gavage	10+14 d	η	η		η	η		τ	τ		η	(185)
Wis	50 mg/kg/d	i.p.	20 d									η		(186)

η, increase; τ, decrease; -, no change; Adm, route of administration; B6, C57BL/6; Wis, Wistar; SD, Sprague Dawley; i.p., intraperitoneal injection; i.v., intra-venous injection; Cr, plasma/serum creatinine; BUN, blood urea nitrogen; UP, urine protein; Fib, fibrosis; Tub/glm, tubular/glomerular damage; BP, blood pressure; EF, ejection fraction; FS, fractional shortening; LVH, left ventricular hypertrophy; C-dam., cardiac damage; d, day; w, week.

**Table V:** Standardized comparison of experimental models used to study CRS.

Model	CRS subtype	Typical timeline	Core renal phenotype	Core cardiac phenotype	Key mechanisms	Best translational use
5/6 Nephrectomy	CRS4	Weeks-months	Progressive CKD, proteinuria, glomerulosclerosis, fibrosis	LVH, myocardial fibrosis, diastolic dysfunction	RAAS activation, inflammation, uremic toxins, oxidative stress	CKD-driven cardiac remodeling and chronic CRS mechanisms
UUO	CRS4-like	Days-weeks	Tubular injury, interstitial inflammation, fibrosis	Cardiac fibrosis and hypertrophy (often mild functional changes)	TGF-β signaling, inflammation, oxidative stress	Renal fibrosis pathways and early kidney-heart signaling
rIR	CRS3	H-days	Acute tubular injury, AKI, inflammation	Myocardial inflammation, impaired contractility	Cytokine release, oxidative stress, mitochondrial dysfunction	Mechanistic study of AKI-induced cardiac injury
Cisplatin-induced AKI	CRS3	Days-weeks	Toxic AKI, tubular necrosis, inflammation	Reduced EF, myocardial apoptosis and fibrosis	Oxidative stress, ER stress, mitochondrial injury	Drug-induced nephroto- xicity and AKI-heart axis
Adenine-induced CKD	CRS4	Weeks-months	Tubulointerstitial fibrosis, CKD, metabolic disturbances	LVH, diastolic dysfunction (HFpEF-like)	FGF23 signaling, oxidative stress, inflammation	CKD-associated cardiovascular remodeling

CRS, cardiorenal syndrome; AKI, acute kidney injury; CKD, chronic kidney disease; UUO, unilateral ureteral obstruction; rIR, renal ischaemia-reperfusion; LVH, left ventricular hypertrophy; EF, ejection fraction; RAAS, renin-angiotensin-aldosterone system; HFpEF, heart failure with preserved ejection fraction.

**Table VI:** Advantages and disadvantages of clinical applicability of renal disease models.

Model	Application/*t*ype	Advantages	Disadvantages	(Refs.)
Nx_5/6_	Primary CKD model	Mimics progressive CKD with hypertension and cardiac remodelling.	Does not fully replicate CKD comorbidities (for example, diabetes).	(51,212)
UUO	Obstructive nephropathy and renal fibrosis	Effective in studying renal fibrosis mechanisms.	Does not fully represent chronic kidney stone disease.	(213,214)
IR	Stenosis, kidney transplantation model	Mimics ischaemic AKI and post-transplant injury.	Recovery differs from human kidney transplant conditions.	(215,216)
Cisplatin	Chemotherapy-induced AKI and cardiotoxicity	Models nephrotoxicity and cardiovascular complications seen in chemotherapy patients.	Acute toxicity effects differ from CKD progression, limiting long-term applicability.	(217,218)
Adenine	CKD with metabolic and CV effects	Induces tubulointerstitial injury and fibrosis; mimics metabolic alterations in CKD.	Causes weight loss and systemic metabolic disturbances not fully reflective of human CKD.	(219,220)

CKD, chronic kidney disease; AKI, acute kidney injury; CV, cardiovascular; Nx_5/6_, 5/6 nephrectomy; UUO, unilateral ureteral obstruction; IR, ischaemia-reperfusion.

## Data Availability

Not applicable.
